# Sordarin bound eEF2 unlocks spontaneous forward and reverse translocation on CrPV IRES

**DOI:** 10.1093/nar/gkad476

**Published:** 2023-06-07

**Authors:** Zheren Ou, Alexey Petrov

**Affiliations:** Department of Biological Sciences, Auburn University, Auburn, AL36849, USA; Department of Biological Sciences, Auburn University, Auburn, AL36849, USA

## Abstract

The Intergenic Region Internal Ribosome Entry Sites (IGR IRESs) of *Discistroviridae* promote protein synthesis without initiation factors, with IRES translocation by elongation factor 2 (eEF2) being the first factor-catalysed reaction. Here, we developed a system that allows for the observation of intersubunit conformation of eukaryotic ribosomes at the single-molecule level by labeling rRNA. We used it to follow translation initiation and subsequent translocation of the cricket paralysis virus IRES (CrPV IRES). We observed that pre-translocation 80S–IRES ribosomes spontaneously exchanged between non-rotated and semi-rotated conformations, but predominantly occupied a semi-rotated conformation. In the presence of eEF2, ribosomes underwent forward and reverse translocation. Both reactions were eEF2 concentration dependent, indicating that eEF2 promoted both forward and reverse translocation. The antifungal, sordarin, stabilizes eEF2 on the ribosome after GTP hydrolysis in an extended conformation. 80S–CrPV IRES–eEF2-sordarin complexes underwent multiple rounds of forward and reverse translocations per eEF2 binding event. In the presence of sordarin, neither GTP hydrolysis nor a phosphate release were required for IRES translocation. Together, these results suggest that in the presence of sordarin, eEF2 promotes the mid and late stages of CrPV IRES translocation by unlocking ribosomal movements, with mid and late stages of translocation being thermally driven.

## Graphical Abstract

**Graphical Abstract:**
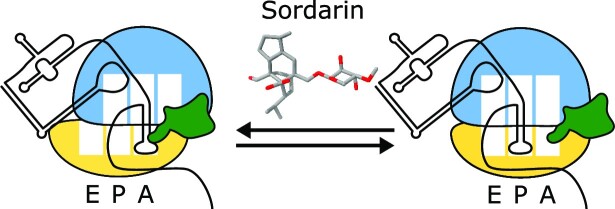

## INTRODUCTION

Viruses employ alternative translation initiation mechanisms to translate their mRNAs. Internal ribosome entry sites (IRESs) are highly structured, untranslated regions of the viral mRNA that promote efficient cap-independent translation initiation (1,2). The intergenic region (IGR) IRESs of Dicistroviruses are the simplest IRESs that start translation in the absence of all initiation factors (3–5). The most studied IGR IRESs are the Cricket paralysis virus (CrPV), Taura syndrome virus (TSV) and Plautia stali intestine virus (PSIV) IRESs, which all initiate through a similar mechanism. It begins with the IRES directly binding to the 40S ribosomal subunit, forming the 40S–IRES complex (4). This complex then recruits the large ribosomal subunit. In the resulting 80S–IRES ribosomes, the IRES occupies the intersubunit space spanning from the A- to E-site, where it interacts with the phylogenetically-conserved 80S core (6–8). Consequently, CrPV IRES is active in a broad range of organisms and can promote translation in insect cells (9,10), mammalian cells and extracts (4,11), and yeast (12,13). CrPV IRES is composed of three structural domains (14). Domains I and II recruit the ribosome (11,15). Domain III, composed of RNA pseudoknot I (PKI) with the GCU translation start codon, is located immediately downstream. In 80S–IRES complexes, PKI occupies the A-site of the ribosome and spans into the decoding center, where it mimics the anticodon stem of tRNA base paired to a mRNA (16,17). Thus, subunit joining results in pre-translocation ribosomes, and the 80S–IRES complexes undergo a translocation in order to move PKI from the A-site into the P-site to, in turn, allow for the first codon decoding (17–20). IRES translocation requires eEF2 and places the first GCU codon into the A-site, where it is decoded by eEF1A-tRNA^Ala^.

Mechanisms of elongation are largely conserved between all kingdoms of life. A majority of our understanding of elongation comes from studies of prokaryotic translation. Peptidyl transfer unlocks the conformational dynamics of the ribosome. After peptidyl transfer, the acceptor ends of the A- and P-site tRNAs move into the P- and E-sites. This results in tRNAs occupying hybrid A/P and P/E states (where the letters before and after the slash designate the position of the tRNA anticodon and acceptor stems, respectively) (21,22). Transition into the hybrid state is concurrent with an ∼9 degrees counterclockwise (forward) rotation of the small subunit relative to the large subunit (22,23). Simultaneously, the L1 stalk moves inward and contacts the elbow of the P-site tRNA (24–26). The pre-translocation ribosomes spontaneously exchange between the classical (A/A and P/P states) and hybrid states (27,28), with the hybrid state being favored at physiological magnesium and polyamine concentrations (29,30). Translocation proceeds via a number of chimeric states, where tRNAs occupy intermediate positions between canonical tRNA binding sites (31–34). A translational GTPase, EF-G, a prokaryotic homolog of eEF2, triggers conformational rearrangements of the ribosome (35), with the ribosomes transiently occupying the rotated state prior to GTP hydrolysis (25,27,36,37). In rotated ribosomes, domain IV of EF-G extends into the A-site and head of the 30S subunit, swivelled by 18 degrees. These rearrangements are accompanied by the movement of the anticodon stem-loop, placing the tRNA into chimeric ap/P and pe/E sites. In the chimeric ap/P state, the anticodon stem-loop of the A-site tRNA moves toward the P-site into the intermediate position between the P- and A-sites, while the acceptor end is located in the P-site. In the pe/E state, the anticodon stem-loop of the P-site tRNA moves closer to the intermediate pe position, while the acceptor end of the tRNA is located in the E-site (31,32,38). Post-GTP hydrolysis, the body of the small subunit undergoes reverse (clockwise) rotation and the head of the small subunit swivels back. The combination of these two motions leads to translocation of the tRNAs into the P- and E-sites (38,39). While translocation can occur in a GTP- and factor-independent manner (40,41), EF-G greatly accelerates translocation, and GTP hydrolysis further increases the rate 10–100 fold (42–44). During translocation, the L1 stalk maintains contact with the tRNA (25,45). In bacteria and higher eukaryotes, after translocation is complete, the L1 stalk moves outward and allows the E-site tRNA to dissociate (25,46,47). However, in fungi, after tRNA movement is complete, the L1 stalk remains in a closed position and its opening is facilitated by elongation factor 3 (eEF3) (48,49).

The translocation of the 80S–IRES complex bears similarity to regular tRNA translocation. The 80S–CrPV IRES pre-translocation complexes occupy non-rotated and semi-rotated conformations that differ by a 5 degrees counterclockwise rotation of the 40S subunit body (17,18). It was proposed that these conformations are at equilibrium that resembles the rotated - non-rotated state exchange in pre-translocation ribosomes. Reminiscent to tRNA translocation, eEF2 binding to 80S–CrPV IRES complexes induces an additional 3 degrees counterclockwise rotation, resulting in fully rotated ribosomes. Simultaneously, tRNA-mRNA mimicking PKI moves into the chimeric ap position on the small subunit (16). The Cryo-EM structure of 80S ribosomes with related TSV IRES (50,51), eEF2 and the antifungal, sordarin, showed ribosomes in five intermediate stages of IRES translocation (52). There, the reverse body rotation and forward head swivel are accompanied by progressive movements of PKI toward the P-site. This movement is followed by a reverse head swivel that coincides with the final placement of PKI in the P-site, yielding a translocated IRES and a non-rotated ribosome (16,52). The translocated 80S–CrPV IRES complex is not stable and back translocates, as was shown by both toeprinting analysis and Cryo-EM (5,53,54). Despite these studies of the 80S–CrPV IRES complex, the molecular mechanisms of IRES initiation and translocation remain unclear.

Eukaryotic translation was previously observed at the single-molecule level. A-site codon decoding and tRNA dynamics before and during translocation in mammalian ribosomes were tracked with tRNA-tRNA FRET (55–57). The FRET between uS19 and uL18 was used to follow subunit joining and dissociation during translation initiation and termination in yeast (58–60). However, intersubunit conformation was not directly probed in these experiments. Here, we report the development and application of a single-molecule FRET system that allows for the observation of intersubunit conformation of *S. cerevisiae* ribosomes in real-time. We used it to follow translation initiation and translocation of CrPV IRES. We showed that 60S subunit joining places ribosomes in both semi-rotated and non-rotated conformations. Pre-translocation 80S–CrPV IRES complexes are exchanging between these two conformations. Using ribosomal rotation as a proxy for translocation, we showed that both forward and reverse translocation are driven by eEF2. GTP hydrolysis is required for rapid forward translocation. However, in the presence of the antifungal sordarin, which stabilizes eEF2 on the ribosome, neither GTP hydrolysis nor a phosphate release were needed, and forward and reverse translocation occurred spontaneously and repeatedly at rates that were comparable with normal translocation. Thus, sordarin captures the ribosome in an unlocked state in which the mid and late steps of IRES translocation are thermally driven. The experiments with GDP and GDPNP indicated that GTP hydrolysis is likely needed to achieve an unlocked state. Together, these results provide support for the Brownian model of translocation.

## MATERIALS AND METHODS

### rRNA mutagenesis, mutant selection and validation

Ribosome mutagenesis was performed, as previously described (61), using the RDN mutagenesis system (62,63). Extended helix 44 of 18S rRNA was amplified from RDN-Ura plasmid containing extended hairpin (61) using F_h44_SexAI and R_h44_MluI primers (Supplementary Table S1). The PCR product was cloned into the RDN operon bearing the pJD180.Trp plasmid (64) by SexAI and MluI sites. Extension of helix 101 of the 25S rRNA was introduced into the plasmid, pJD180.Trp, by two-step megaprimer PCR. First, primers F_h101_b and R_h101.3_b were used to add the 5′ extension and amplify the region downstream of helix 101. Primers F_h101.3_a and R_h101_a were used to introduce the 3′ extension and amplify the region upstream of helix 101. In the second step, upstream and downstream PCR products were annealed together to get the full helix 101 extension. The insertion-bearing hairpin (called sp22) was then cloned into pJD180.Trp by BamHI and MluI sites. The resulting plasmids, pAL783 (pJD180.Trp.h44.sp68) and pAL797 (pJD180.Trp.h101.3.sp22), were transformed into an AL14 *S. cerevisiae* strain (61), which expressed mutant rRNA from pJD694 (URA3) plasmid. Transformants were initially selected on − Trp medium, followed by two passages of replica plating onto 5-FOA medium. RDN mutagenesis and lack of wild type loci were confirmed by RDN PCR and sequencing. A pair of primers, which are about 150 bp up and downstream of the insertion site, were used to amplify the insertion region. The product is 383 bp for the helix 44 mutant and 336 bp for the helix 101 mutant, which are 19 bp and 21 bp longer than the PCR product from the wildtype RDN operon. The PCR product was analyzed by electrophoresis on 4% agarose gel, which migrated as a single band with a size corresponding to the mutant hairpin. Furthermore, sequencing of the PCR product confirmed the presence of the mutant hairpin and absence of wildtype contamination. All strains used and generated in this study are listed in the Supplementary Table S6.

### Purification and labeling of ribosomal subunits

Ribosomal subunits were purified and labeled, as previously described (61,65), with the following modifications: A 5 ml liquid YPAD culture was inoculated with a single colony from a fresh YPAD plate and grown at 250 rpm in a 30°C shaker until OD_600_ = 0.8–1.0. 1 ml of the resulting culture was used to inoculate 1 l of YPAD media. Cells were grown until OD_600_ = 0.8–1.0, harvested by centrifugation, and washed twice with ice-cold buffer A (30 mM HEPES–KOH pH 7.4, 100 mM KCl, 15 mM MgCl_2_, 2 mM DTT). Cells were lysed by a bead-beater and the lysate was cleared by centrifugating twice in an A27-8 × 50 rotor for 15 min at 15 000 rpm. Fines were removed by passing the lysate through a 0.45 μm filter. The lysate was overlayed on a 3 ml cushion of buffer B (30 mM HEPES–KOH pH 7.4, 100 mM KCl, 12 mM MgCl_2_, 2 mM DTT), supplemented with 1 M sucrose and spun down in a Type 70Ti rotor for 5 h at 55 000 rpm. The ribosome pellet was resuspended in buffer B and loaded on top of 5–47% sucrose gradients in buffer B. Gradients were centrifuged at 22 000 rpm for 12 h in a SW32 rotor. Gradients were fractionated to collect 80S ribosomes. Resulting ribosomes were buffer exchanged into buffer B supplemented with 250 mM sucrose, aliquoted and frozen with liquid nitrogen. Purified 80S ribosomes were labeled with corresponding oligonucleotide dye at a 1:2 molar ratio by sequentially incubating at 42°C for 1 min, 37°C for 10 min and 30°C for 10 min. The reaction was loaded on top of 10–30% sucrose gradients (30 mM HEPES–KOH pH 7.4, 500 mM KCl, 7.5 mM MgCl_2_, 2 mM DTT supplement with sucrose) and centrifugated at 24 000 rpm for 12 h in a SW32 rotor. Gradients were fractionated and 40S and 60S ribosomal subunits were collected. Labeled ribosomal subunits were then concentrated and the buffer was exchanged to a storage buffer (30 mM HEPES–KOH pH 7.4, 100 mM KCl, 5 mM MgCl_2_, 2 mM DTT and 250 mM sucrose) using Amicon^®^ Ultra-15 100 kDa centrifugal filters. Ribosomal subunits were aliquoted and frozen with liquid nitrogen. The purity and integrity of the subunits were validated by composite agarose-acrylamide gel electrophoresis. The labeling efficiency was measured spectrophotometrically and was found to be 92% and 88% for 40S and 60S subunits, respectively. The measurement was confirmed by quantifying the fluorescence intensity of the 40S-Cy3B and 60S-Cy5 subunit bands of the composite gel.

### Purification of elongation factors

eEF1A, eEF1Bα, eEF2 and eEF3 were purified, as previously described (65–68).

#### eEF1A

Untagged eEF1A was purified from the *S. cerevisiae* strain, CB010, as previously described (66) with the following modifications: The lysate was clarified by centrifugation in an A27-8 × 50 rotor at 15 000 rpm for 30 min, followed by centrifugation in a Type 70 Ti rotor at 50 000 rpm for 90 min. The clarified lysate was applied to a gravity-flow 7 ml DEAE column equilibrated with eEF1A buffer A (20 mM Tris–HCl, pH 7.5, 100 mM KCl, 0.1 mM EDTA, 1 mM DTT, 25% glycerol). After loading the lysate, the column was washed with two column volumes (CV) of eEF1A buffer A. The flow-through and column wash were pooled and loaded on a gravity-flow 5 ml SP Sepharose column equilibrated with eEF1A buffer A. The SP Sepharose column was washed with 5 CV of eEF1A buffer A before eluting. eEF1A was eluted with eEF1A buffer 150B (eEF1A buffer A with 150 mM KCl) for 5 CV at first, then eluted with buffers 250B, 350B and 500B for 4 CV. 5 ml fractions were collected. eEF1A-containing fractions were pooled and were concentrated to ∼5 ml. The concentrated protein was applied to a 26/600 Superdex 75 size exclusion column equilibrated with a storage buffer (20 mM Tris–HCl, pH 7.5, 100 mM KCl, 0.1 mM EDTA, 1 mM DTT, 25% glycerol). Fractions containing pure eEF1A were identified by SDS-PAGE, pooled, and concentrated using Amicon^®^ Ultra-15 30 kDa MWCO centrifugal filter units.

#### eEF1Bα

The pET28b plasmid, carrying eEF1Bα-TEV site-His6, was transformed into BL21-RIPL *Escherichia coli* cells. The protein was expressed and purified, as previously described (65).

#### eEF2

6× His tagged eEF2 was purified from the *S. cerevisiae* strain, TKY 675, as previously described (67), with the following modifications. Cells were grown in YPAD at 30°C to OD_600_ = 1.5 and harvested by centrifugation. The cells were resuspended with ice-cold lysis buffer (50 mM potassium phosphate, pH 7.6, 300 mM KCl, 1 mM DTT, 10 mM imidazole) and lysed using a bead-beater. After lysis, the pH of the lysate was adjusted to 7.7 with 1 M un-titrated Tris and clarified by centrifugation in an A27-8 × 50 rotor at 12500 rpm for 20 min. The resultant supernatant was centrifuged at 50 000 rpm in a Type 70 Ti rotor for 90 min. The lysate was passed through a 0.22 μm filter and loaded onto a 5 ml HisTrap HP column (GE Healthcare). The column was washed with 25 ml of lysis buffer containing 30 mM imidazole. Proteins were eluted with lysis buffer containing 250 mM imidazole. The eluate was buffer exchanged into buffer A (20 mM Tris–HCl pH 7.6, 30 mM KCl, 5 mM MgCl_2_, 1 mM DTT). Protein was loaded onto a 5 ml HiTrap Q column. Column was washed with 6 CV of buffer A. Proteins were eluted with a 15 CV gradient of 30–500 mM KCl. Fractions containing eEF2 were pooled and concentrated using an Amicon^®^ Ultra-15 30 kDa MWCO centrifugal filter and buffer exchanged into storage buffer (20 mM Tris–HCl, pH 7.5, 100 mM KCl, 0.1mM EDTA, 1 mM DTT, 25% glycerol).

#### eEF3

6× His tagged eEF3 was purified from the *S. cerevisiae* strain, TKY 1653, as previously described (68).

### Preparation and biotinylation of CrPV IRES

CrPV IRES was prepared by T7 runoff transcription. The template plasmid, T7-IGR-CrPV-IRES (65), bears a 6025–6232 nts region of CrPV genome that contains a CrPV IGR IRES located at the 6030–6219 genomic coordinates. The plasmid was linearized with NarI and transcribed with NEB HiScribe™ T7 High Yield RNA Synthesis Kit. The resulted RNA was purified by gel filtration on a SEC650 column, as described before (69). RNA was concentrated using a 30 kDa MWCO spin concentrator. The 5′-biotinylated DNA oligonucleotide was annealed to the 3′ end of the RNA to a region located 39–54 nts downstream of pseudoknot I of CrPV IRES, as described before (65).

### Single-molecule imaging and data analysis

A home-built total internal refection fluorescence (TIRF) microscope was used for single-molecule imaging. The prism-based system was built on the base of a Nikon Ti2-A inverted microscope. The viewing area of ∼3000 μm^2^ (∼ 55 × 55 μm) was illuminated with a 532 nm continuous wave fiber laser (MPB Communications). The excitation laser was spectrally cleaned up with 532/20 nm bandpass filter. The laser power was 50 mW for 100 ms imaging and 150 mW for 25 ms imaging. The sample was imaged with a CFI Plan Apo 60×, N.A. = 1.2, water immersion objective. Cy3B and Cy5 fluorescence was separated using Photometric Quad View with a 640 long pass dichroic mirror and imaged in the 585/70 and 700/75 channels for Cy3B and Cy5 dyes, respectively. All filters and mirrors were obtained from Chroma Technology. Non-binned images were recorded using an Andor iXon Ultra 897 EMCCD camera with a 600 EM gain and no pre-gain. The solvent delivery system was built using a J-Kem syringe pump equipped with a 100 μl syringe. The imaging chambers were constructed out of a quartz slide, double-sided tape, and a coverslip. The chamber volume was approximately 8 μl. Both slides and coverslips were treated with PEG 5000 and PEG-biotin 5000, thus passivating the surface and providing molecular handles for immobilization (28). PEG-Biotin-treated quartz slides were washed twice with 200 μl of TP50 buffer (50 mM Tris–HCl, pH 7.5, 100 mM KCl) and incubated with 1 μM neutravidin, 0.67 mg/ml BSA, and 1.3 μM of blocking DNA oligonucleotide duplex for 5 min. Slides were washed twice with 200 μl of TP50 and one time with a reaction buffer containing 30 mM HEPES–KOH, pH 7.4, 100 mM KCl, 5 mM MgCl_2_ and 1 mM Spermidine. 40S–CrPV IRES complexes were prepared by incubating 100 nM of 40S-Cy3B fluorescent subunits and 100 nM CrPV IRES-biotin in a reaction buffer at 30°C for 5 min. 40S–CrPV IRES complexes were diluted to 0.8 nM with the reaction buffer and immobilized for 5 min. Non-immobilized ribosomes were removed by washing the chambers with reaction buffer. Finally, the chambers were washed with the reaction buffer supplemented with 1mM GTP, 2 mM TSY, and an oxygen scavenging system composed of 2.5 mM protocatechuic acid and 0.06 U/μl protocatechuate dehydrogenase (70). A 50 μl delivery mix was flowed to the slide simultaneously with the start of observation. For experiments with sordarin (Cayman Chemical #26255), the drug concentration was 20 μM. All experiments were performed at 21°C.

Fluorescent traces were extracted using home-written MATLAB scripts. Time-dependent donor and acceptor fluorescence intensities were converted into FRET efficiency using the formula, FRET = *I*_acceptor_/(*I*_acceptor_ + *I*_donor_). Individual molecules were identified by single step photobleaching events. FRET trajectories were idealized using ebFRET software (71,72). According to ELBO criterion, the three-state model (two FRET states and FRET off) was preferable in most experiments. However, the minority of traces (∼1%) clearly showed three distinct FRET states. Unfortunately, signal-to-noise hampered the identification of this third state in the majority of molecules. Therefore, to avoid overinterpretation of the results, we selected a simpler two-FRET-state model.

All statistical analysis was performed in MATLAB. Kinetic parameters were extracted by building a cumulative probability plot and fitting observed data to linear, single-, or double-exponential functions. The reported 95% confidence interval is a goodness of fit. The rotating molecules were defined as molecules with at least two unambiguous FRET transitions between the fitted FRET states. All related data size and statistical parameters were reported in Supplementary Tables S2–S5.

## RESULTS

### Following 80S–CrPV IRES complex formation in real-time

**Figure 1.**
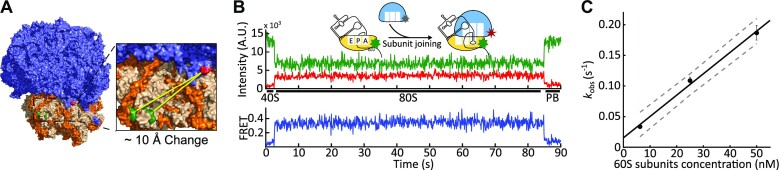
Following 80S–CrPV IRES formation in real-time. (**A**) 40S (orange) and 60S (blue) yeast ribosomal subunits were labeled at h44 (green) and h101 (red), respectively. The distance between dye positions is expected to change by ∼10 Å when the ribosome transits between the rotated and non-rotated conformations. Based on PDB IDs, 3J77 and 3J78 (74). (**B**) FRET between h44 and h101 attached dyes was used to follow 60S subunit joining to 40S–IRES complexes. The 40S–IRES complexes were immobilized on the slide and 60S subunits were delivered concurrently with the start of observation. 80S ribosome formation was detected by an appearance of FRET (∼3 s in the example trace). FRET remained stable, as framerate is insufficient to detect spontaneous rotations. (**C**) 60S subunit arrival rate was concentration dependent. The arrival rate constant was measured as a slope of the concentration dependence curve and was determined to be 3.5 ± 1.0 μM^−1^ s^−1^. Error bars are the errors of the exponential fits (*n* = 102, 130 and 117 molecules for 6.25, 25 and 50 nM 60S ribosomal subunits, respectively).

To follow the conformation of eukaryotic ribosomes in real-time, we site-specifically labeled yeast small and large ribosomal subunits with fluorescent dyes. Phylogenetically variable loops of helix 44 of 18S rRNA and helix 101 of 25S rRNA were extended with distinct weak hairpins (Figure 1A). Isolated strains were confirmed to have only mutant rDNA by PCR and RDN loci sequencing (Supplementary Figure S1). Low thermodynamic stability of the inserted hairpins allows for site-specific annealing of fluorescent DNA oligonucleotides at physiological temperatures (61,73). Small ribosomal subunits were site-specifically labeled with Cy3B dye and large ribosomal subunits were labeled with Cy5 dye by annealing fluorescently labeled DNA oligonucleotides to the h44 and h101 extensions, respectively (Supplementary Figure S1).

The Cryo-EM structures of both 80S ribosomes, in complex with tRNAs and IGR IRESs, show that the apical positions of h44 and h101 are within FRET distance and are ∼60 Å apart in the non-rotated conformation (18,52,74). The distance between dyes increases by ∼10 Å when the ribosome transitions from the non-rotated to rotated conformation (PDB IDs: 3J77 and 3J78) (74). This distance between dyes and FRET efficiency are anticorrelated. This allows us to follow 60S subunit joining by the appearance of FRET and ribosome conformation by measuring FRET efficiency.

Using this system, we first followed 80S ribosome assembly on CrPV IRES. The 40S–CrPV IRES complex is exceedingly stable with a lifetime of more than 400 s (65). This allowed us to prepare and surface-immobilize 40S-Cy3B–CrPV IRES complexes using biotinylated CrPV IRES. Then, Cy5-labeled large ribosomal subunits were delivered simultaneously with the start of observations. 80S ribosome formation was detected by an appearance of FRET (Figure 1B). 60S ribosomal subunit binding was efficient, with 70% of the 40S–IRES ribosomes forming 80S–IRES complexes. The large subunit arrival rate was fast, with *k*_obs_ = 0.19 ± 0.012 s^−1^ at 50 nM 60S subunits (Supplementary Table S2). Consistent with bimolecular reaction, the arrival rate was concentration dependent (Supplementary Figure S2A). The rate constant was determined as a slope of concentration dependence and was found to be 3.5 ± 1.0 μM^−1^s^−1^ (Figure 1C). The measured rate is comparable with 60S subunit recruitment rates during factor-driven initiation measured by ensemble and single-molecule approaches (0.076 and 0.2 s^−1^, respectively, at 100 nM 60S ribosomal subunits (59,75)). Consistent with previous studies (65), 80S–CrPV IRES complexes were stable, with lifetimes longer than the five minute observation window (Supplementary Figure S2B). Thus, labeled ribosomal subunits are active in IRES-driven initiation.

### Spontaneous rotations in pre-translocation 80S–CrPV IRES complexes

**Figure 2.**
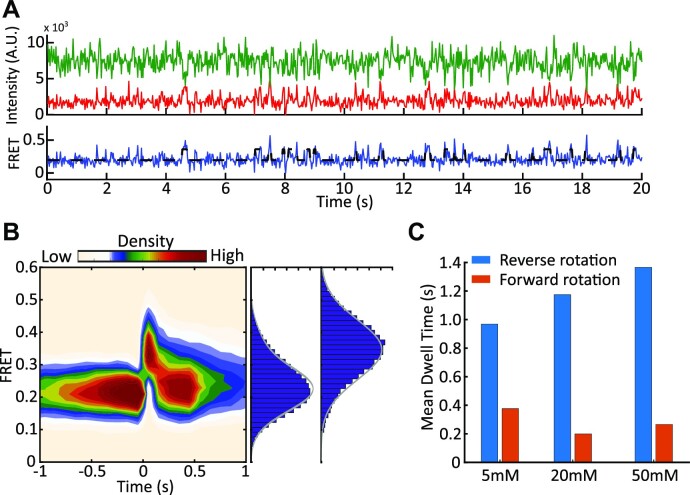
Spontaneous rotation of pre-translocation 80S–CrPV IRES complexes. (**A**) Example trace showing spontaneous rotations of the 80S–CrPV IRES complex. (**B**) Density heatmap. FRET traces were post-synchronized to the point where semi-rotated ribosomes transited to the non-rotated state. The synchronization point was set as time 0. The FRET distributions for semi-rotated ribosomes and non-rotated ribosomes were best described by a single Gaussian. The estimated mean FRET efficiency was 0.22 ± 0.08 (95% CI) for the semi-rotated state and 0.34 ± 0.08 (95% CI) for the high FRET state. (**C**) Bar chart of mean dwell times for reverse (blue) and forward (orange) rotations at various Mg^2+^ concentrations at 25 ms exposure time.

The Cryo-EM structures show 80S–IRES complexes in both semi-rotated and non-rotated conformations. It was proposed that 80S–IRES complexes are spontaneously exchanging between these conformations (17,18). In the experiments described above, ribosomes predominantly occupied the low, ∼0.22, FRET state and transiently transitioned into the higher, ∼0.34, FRET state (Figure 2B). Cryo-EM structures of 80S IRES complexes showed that the distance between the end of helix 44 and the end of helix 101 decreases by ∼5 Å as the ribosome changes from the semi-rotated to the non-rotated conformation (Supplementary Figure S3) (17,18). Thus, we assigned the low (0.22) FRET state to the semi-rotated ribosomes and the high (0.34) FRET state to the non-rotated ribosomes. Consistent with two conformations, a total FRET efficiency histogram of 80S–IRES complexes had a right shoulder and was best approximated by a double Gaussian fit. The FRET states of the double Gaussian fit matched individual FRET distributions and had an efficiency of 0.2 ± 0.06 (95% CI) for the low FRET state and 0.33 ± 0.07 (95% CI) for the high FRET state (Supplementary Figure S2C).

The experiments described above were done at a 100 ms exposure time. At this condition, 27% of 80S ribosomes showed spontaneous rotations, with an average of 2.3 rotations per trace (average FRET duration is 169 ± 10.3 s) (Supplementary Table S2). This is substantially less frequent than what was observed in pre-translocation bacterial and human ribosomes (27,76). Importantly, spontaneous transitions of pre-translocation ribosomes in complex with tRNAs, are fast and occur on the sub-second timescale (27,28,77–79). We hypothesized that spontaneous transitions (and particularly the transient high state) are faster than 100 ms, and, thus, escape detection due to time-averaging by the imaging camera (80). To determine if the low frame rate masked spontaneous transitions, we imaged 80S–CrPV IRES complexes at a 25 ms resolution. The increased time resolution made delivery experiments challenging, due to a lower signal-to-noise ratio (SNR) caused by lower photon budget and SNR decrease due to mechanical stresses produced by the solvent delivery system. Therefore, 25 ms experiments were done with pre-formed 80S–CrPV IRES complexes. There, 83% of pre-translocation ribosomes underwent spontaneous rotations with an average of 23.3 rotations per molecule (Supplementary Table S2). The dwell times were best described by a double exponential function (Supplementary Figure S4), indicating kinetic heterogeneity. Ribosomes highly preferred the semi-rotated state, with a mean dwell time being 0.97 s, while the non-rotated state was approximately three times shorter at 0.38 s (Figure 2C). The median high FRET state dwell time was 0.05 s, which explains why spontaneous rotations were rarely observed at 100 ms exposure time and confirms that they have been masked by low framerate (Supplementary Table S2). Together, this confirms the hypothesis that 80S–IRES complexes spontaneously exchange between semi-rotated and non-rotated conformations.

### eEF2 facilitates intersubunit dynamics

Translocation is accompanied by a reverse subunit rotation and results in non-rotated ribosomes. Translocation of 80S–tRNA complexes is unidirectional and results in stable translocated ribosomes. However, translocated IRES complexes are unstable and back-translocate (17,54). To follow translocation of CrPV IRES, we co-delivered 60S subunits, eEF2, and GTP to the immobilized 40S–CrPV IRES complexes. Spontaneous rotations complicate identifications of conformational transitions associated with translocation. Thus, we conducted these experiments at 100 ms time resolution, so that spontaneous transitions in the pre-translocation complexes are masked by camera averaging and predominantly eEF2-induced rotations are visible.

**Figure 3.**
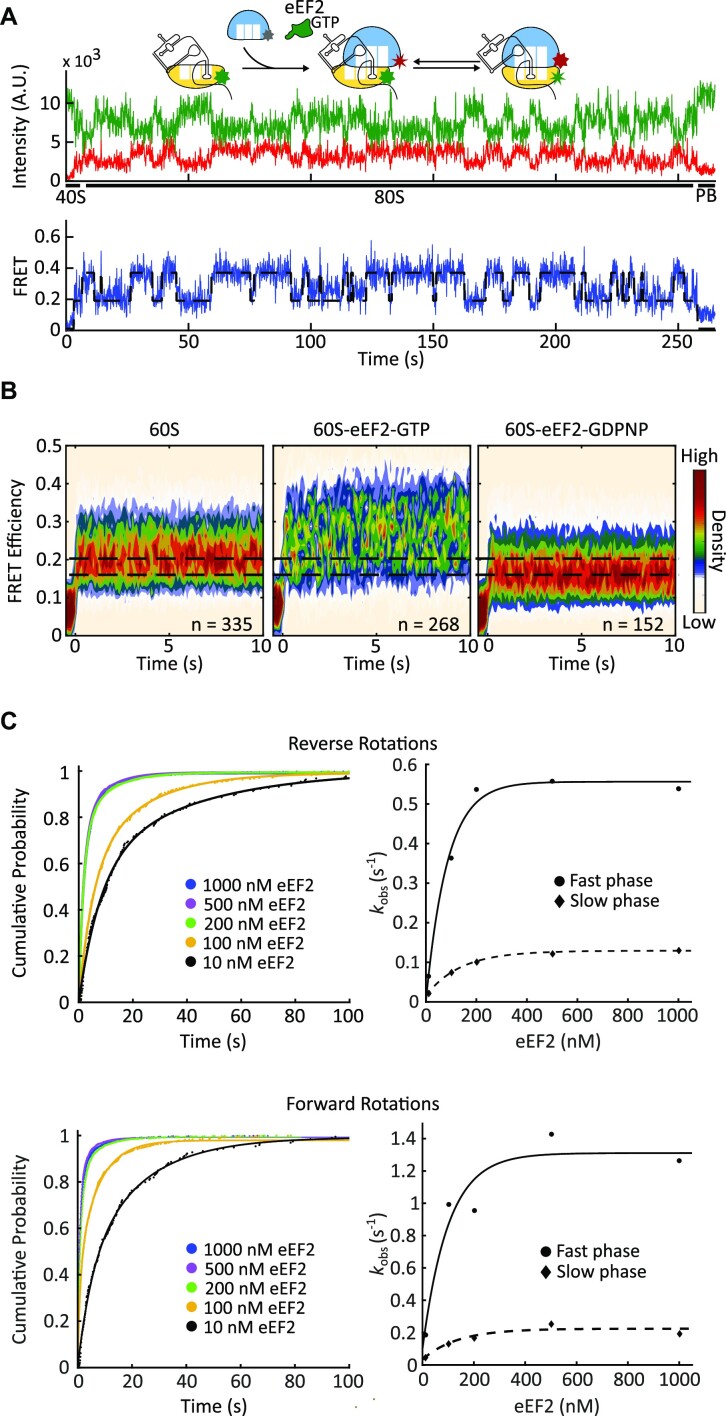
eEF2 facilitates intersubunit rotations. (**A**) Example trace of 80S–CrPV assembly in the presence of eEF2-GTP. 40S–IRES complexes were immobilized to the slide and 25 nM 60S subunits, eEF2 at various concentrations, and 1 mM GTP were co-delivered concurrently with the start of observation. Upon subunit joining, FRET oscillated between high and low FRET states that correspond to the non-rotated and semi-rotated ribosomal conformations. (**B**) Density heatmap of FRET traces. FRET traces were post-synchronized to the beginning of FRET (60S subunit binding), with the synchronization point being set to time 0. In the presence of GTP, ribosomes fluctuated between semi-rotated and non-rotated conformations. Ribosomes rapidly shifted into the rotated state in the presence of GDPNP. (**C**) Cumulative probability plot of reverse (top) and forward rotation (bottom) dwell times with different eEF2-GTP concentrations. Rotation rates saturated at around 200 nM eEF2. Lines represent double-exponential fitting (left) and the resulted rates were fit by hyperbolic functions (right panel. Solid line for the fast phase, dashed line for the slow phase) (*n* = 143, 129, 268, 202 and 169 for 10, 100, 200, 500 and 1000 nM, respectively).

In the presence of eEF2-GTP, the 80S ribosomes began exchanging between the semi-rotated (0.23) and non-rotated (0.34) FRET states (Figure 3A) at rates that are drastically different from spontaneous rotations (Figure 2C, Supplementary Figure S5A). The semi-rotated state mean dwell time was 8.4 s at 100 nM eEF2 and 0.97 s in the absence of eEF2. The non-rotated state mean dwell time was 5.9 s at 100 nM eEF2 and 0.38 s in the absence of eEF2, indicating that these transitions were eEF2-induced (Supplementary Tables S2 and S3). Translocation places the ribosome into the non-rotated, high FRET state. Thus, a low-to-high FRET transition, in the presence of eEF2, corresponds to the clockwise intersubunit rotation and forward translocation. By extension, a high-to-low FRET transition corresponds to the counterclockwise rotation, and back translocation.

To understand the translocation mechanism, we varied eEF2 concentration in the delivery mix from 10 to 1000 nM. The rates of both forward translocation (low-to-high FRET) and, surprisingly, reverse (high-to-low FRET) translocation were dependent on eEF2 concentration, indicating a bimolecular mechanism. The kinetics of both reactions were remarkably similar. Both were best approximated by double exponential fits. The rates of both fast and slow components increased with eEF2 concentrations, while relative amplitude increased for the fast component and decreased for the slow component (Supplementary Table S3). Both fast and slow components saturated at ∼200 nM eEF2 for both the forward and reverse rotations (Figure 3C). Interestingly, this is comparable with the previously measured *K*_m_ of GTP hydrolysis by EF-G (80 nM (81) to 400 nM (82)). Thus, eEF2 efficiently engages both conformations of the 80S–IRES complexes and promotes both forward and reverse translocation. The nature of both phases remains to be delineated. In these experiments, we are observing multiple cycles of translocation. Presumably, eEF2 dissociates and rebinds after every reaction, which makes this a multistep process. Such behavior might potentially result in double exponential kinetics. Furthermore, both fast and slow phases are saturated at 1 and 0.2 s^−1^, which is substantially slower than tRNA translocation (83), indicating that IRES imposes additional barriers for translocation that may be surpassed in a multistep fashion. An insufficient imaging framerate might also lead to the kinetic heterogeneity (80,84). Lastly, ribosomal complexes are likely not uniform, as shown by kinetic heterogeneity of spontaneous intersubunit rotations, potentially leading to double exponential kinetics of translocation.

As a control, we replaced GTP with GDP and the non-hydrolyzable GTP analog, GDPNP. eEF2-GDPNP stabilizes ribosomes in the fully rotated state, characterized by an additional 3° counterclockwise rotation (16) that should further decrease the FRET efficiency. In accordance with this, 80S–IRES–eEF2–GDPNP complexes had a lower FRET of 0.17 ± 0.04 (Figure 3B, Supplementary Table S5) that corresponds to the fully rotated ribosomes. Translocation, in the presence of GDP, is slow due to a low affinity of eEF2-GDP to the ribosome and the absence of GTP hydrolysis (42,43). Here, eEF2-GDP had a modest effect on ribosome conformation (Supplementary Figure S6). It slightly increased the number of rotated to non-rotated state transitions, but did not result in stably translocated ribosomes, potentially due to a limited observation time.

### Sordarin bound eEF2 accelerates intersubunit rotation

**Figure 4.**
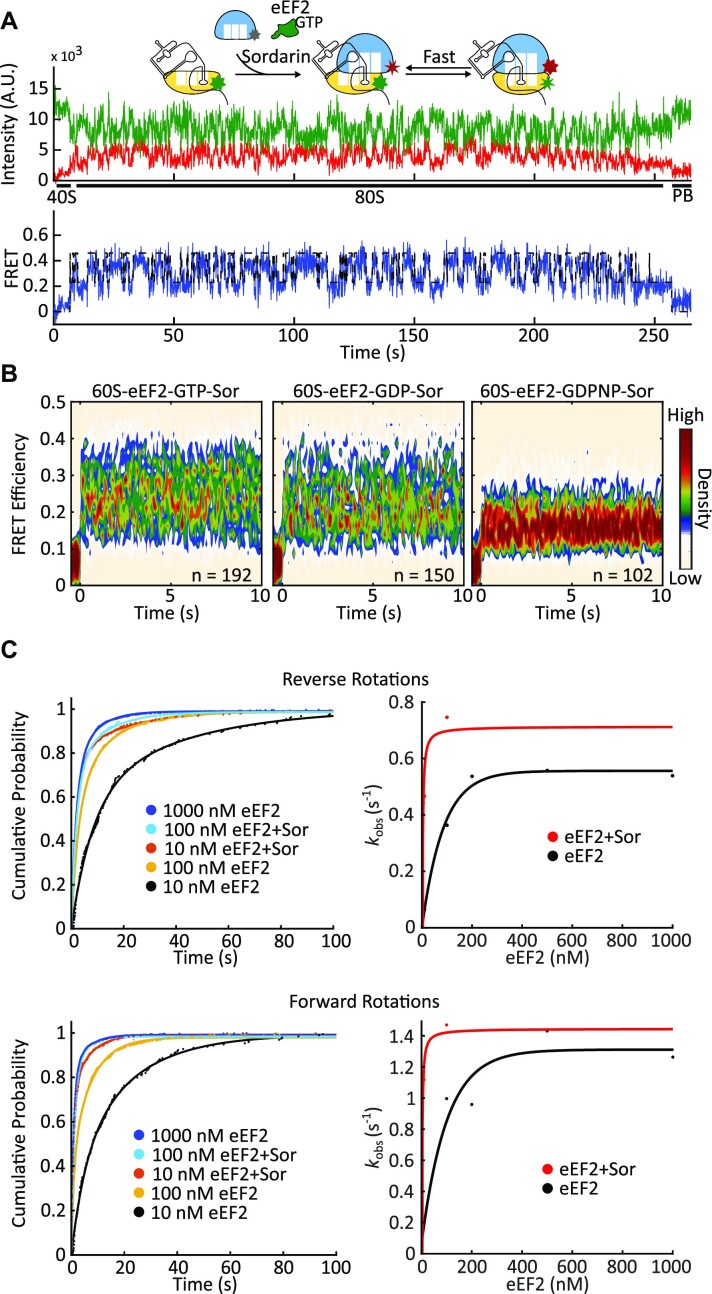
Intersubunit rotations are accelerated in the presence of sordarin. (**A**) Example experimental trace of the dynamic behaviour of the 80S–CrPV–eEF2–GTP–Sordarin complex. 40S–IRES complexes were immobilized on the slide and 60S subunits, eEF2, sordarin, and GTP were co-delivered concurrently with the start of observation. Ribosomes continuously oscillated between the non-rotated and rotated states. (**B**) Density heatmap of FRET traces. FRET traces were synchronized with respect to the beginning of FRET (binding of the 60S subunit). The synchronization point was set at time 0. In the presence of sordarin and GTP, as well as sordarin and GDP, ribosomes fluctuated between semi-rotated and non-rotated conformations. However, ribosomes were mostly stable in the rotated state in the presence of eEF2-GDPNP. This is consistent with sordarin acting post-GTP hydrolysis and binding to eEF2 in the post-GTP hydrolysis state. (**C**) Cumulative probability plots (left panels) show the comparison of reverse (top) and forward rotation (bottom) dwell times with and without sordarin. Rotations saturated at around 10 nM eEF2 in the presence of sordarin and at 200 nM eEF2 without. The lines represent a double-exponential fit (left panels). Resulted fast phase rates were fit by hyperbolic functions (red- with sordarin; black- without Sordarin, right panels).

Sordarins are a class of tetracyclic diterpene glycosides that specifically inhibit fungal protein synthesis, but do not affect bacterial, plant, or mammalian translation (85–87). Sordarin acts by stabilizing eEF2 on the ribosome (86). It does not inhibit GTP hydrolysis and does not affect phosphate release (52,86). It binds to the lever arm of eEF2 in the cleft between domains III, IV and V and restricts compaction of eEF2, thus stabilizing eEF2 in the extended conformation and preventing eEF2 dissociation (85,88). We used sordarin to probe the role of eEF2 in IRES translocation. First, we followed 80S ribosome formation in the presence of sordarin by delivering 60S ribosomal subunits and sordarin (no eEF2) to 40S–CrPV IRES complexes. As expected, sordarin alone did not affect 80S complex formation or spontaneous rotations in pre-translocation 80S–IRES complexes (Supplementary Table S5). Then, we repeated these experiments with sordarin and eEF2. Similar to the experiments without the drug, eEF2-soradarin promoted intersubunit rotations (i.e. forward and reverse translocation) (Figure 4A). However, reaction rates were greatly increased at low eEF2 concentrations. Furthermore, the saturation concertation of eEF2 was 20 times lower in the presence of sordarin than in the absence of the drug (10 nM vs 200 nM eEF2) (Figures 4C, Supplementary Table S4). Together, with previous reports showing that sordarin stabilizes eEF2 on the ribosome, this suggests that when sordarin is present, eEF2 promotes multiple rounds of forward and reverse rotations without dissociating from the ribosome.

The Cryo-EM structure of 80S ribosomes with TSV IRES, eEF2 and sordarin provide us with structural information needed to connect ribosomal rotations to the functional states of the ribosome in the presence of sordarin. As shown in the 80S–TSV IRES–eEF2–sordarin complex structure, the degree of subunit rotation correlates with the position of PKI (Supplementary Figure S3), which shows a gradual movement of PKI between the A- and P-sites as the ribosome rotates. Overall, an un-translocated 80S–IRES complex, characterized by the rotated conformation, transitions into a fully translocated complex, characterized by the non-rotated conformation. This implies that the subunit rotations of the 80S–TSV IRES–eEF2–sordarin complex are correlated with IRES translocation (52). We used this relationship between IRES translocation and subunit rotations in our data interpretation. In our experiments with sordarin and eEF2, a clockwise rotation always corresponds to forward translocation and a counterclockwise rotation corresponds to reverse translocation. Thus, in the presence of sordarin, 80S–CrPV IRES–eEF2 complexes continuously translocate and back translocate.

**Figure 5.**
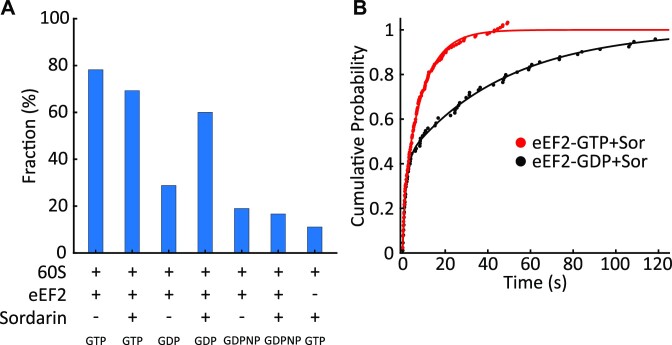
GTP hydrolysis is not required for rotations in the presence of sordarin. (**A**) A fraction of molecules that fluctuated between rotational states (*n* = 202, 192, 163, 150, 152, 102 and 108, respectively). GDPNP suppresses ribosomal rotations in the absence and presence of sordarin, consistent with sordarin acting post-hydrolysis. Rotations were common with GTP and GDP in the presence of sordarin, indicating that energy from GTP hydrolysis was not required. Thus, GTP hydrolysis is needed to achieve conformations in which rotations are unlocked, but it is not needed for subsequent transitions. (**B**) The dwell times before the first rotations were fitted by a double exponential function. The first rotation is slower with GDP, consistent with a decreased eEF2-GDP affinity to the ribosome.

EF-G, a prokaryotic analog of eEF2, cannot efficiently exchange nucleotides while being ribosome-bound (42), suggesting that in the presence of sordarin, the energy of GTP hydrolysis is not required for repeated rounds of forward and reverse translocation. To determine if GTP hydrolysis provides energy to IRES translocation, we repeated experiments with GDPNP and GDP. In the presence of sordarin, more than 80% of 80S–IRES–eEF2–GDPNP complexes were characterized by stable FRET (Figure 5A, Supplementary Table S5), which is consistent with the previous proposal that sordarin acts post-GTP hydrolysis (45,60). On the other hand, eEF2-sordarin promoted subunit rotations in the presence of GDP (Figures 4B and 5A). The first conformational transition in the presence of GDP and sordarin was slightly slower, taking 8.4 s (versus 6 s in the presence of GTP), while the subsequent transitions were indistinguishable from transitions in the presence of GTP (Figure 5B, Supplementary Figure S7). Thus, in the presence of sordarin, GTP hydrolysis is not required for repeated rounds of forward and reverse translocation.

**Figure 6.**
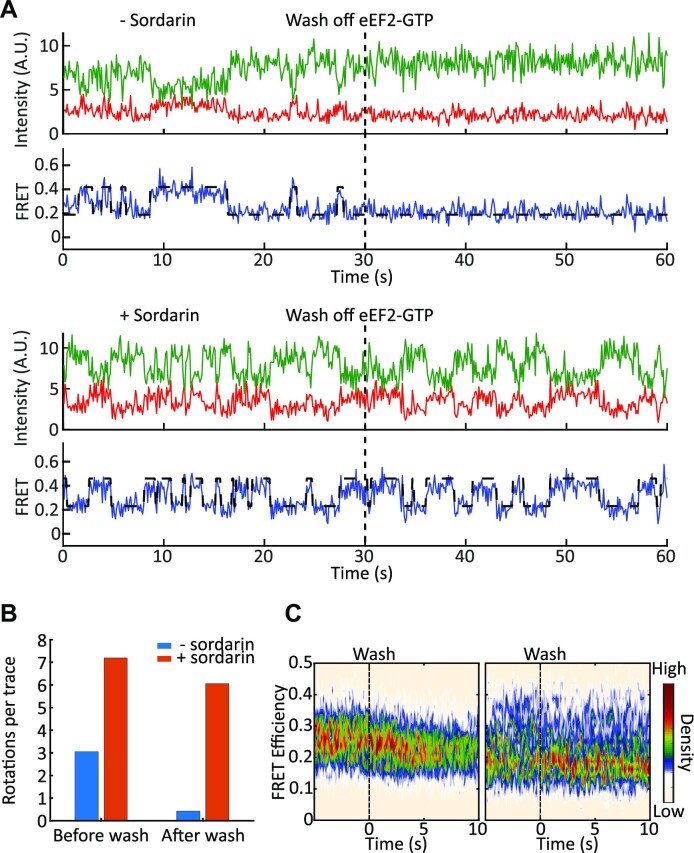
Intersubunit rotations in the 80S–CrPV IRES–eEF2 complex are thermally driven in the presence of sordarin. (**A**) Example traces from a wash off experiment. 80S–CrPV IRES complexes were immobilized to the slide with 100 nM eEF2-GTP without (top) and with (bottom) sordarin (*n* = 142 and 126, respectively). The ribosome oscillates between the non-rotated and rotated states before the wash. After the wash, rotations disappear in the experiment without sordarin, but the ribosome continues fluctuating in the presence of sordarin. (**B**) Number of transitions before and after the wash for eEF2 (blue) and eEF2 plus sordarin (red). (**C**) Traces were synchronized with the wash time set to zero. Ribosomes preincubated with eEF2 (left) rapidly settled in the low FRET state, while ribosomes preincubated with eEF2 and sordarin (right) experienced multiple rounds of rotations.

To exclude the possibility that eEF2 rebinding is driving these events, we conducted wash-off experiments. In these experiments, immobilized 80S–IRES ribosomes were preincubated with eEF2-GTP, either in the presence or absence of sordarin, and immobilized on the surface of a microscope slide. Ribosomes were then imaged to confirm the expected behavior. After 30 s of imaging, eEF2 and nucleotides were replaced with buffer that did not contain eEF2 and GTP and observation continued. Prewash imaging served as a control and showed that ribosomes underwent rotations identical to ones seen in the experiments described above. After the wash-off, control ribosomes (preincubated without sordarin) showed stable FRET with an average of 0.4 rotations per ribosome (in a 30 s observation window after the wash). However, ribosomes that were preincubated with sordarin continued to fluctuate between high and low FRET states, with an average of 6.1 FRET transitions per ribosome (Figure 6, Supplementary Table S5). Because the wash buffer did not contain eEF2 and GTP, the wash precluded the possibility of eEF2 rebinding to the 80S–IRES complexes or nucleotide exchange. Therefore, repeated rounds of translocation are not caused by eEF2 rebinding, but rather promoted by stably bound eEF2 and are thermally driven. This interpretation is also consistent with faster translocation rates in the presence of sordarin, where the reaction essentially becomes unimolecular and, thus, is expected to be faster than bimolecular translocation in the absence of the drug.

### eEF3 does not affect translocation of CrPV IRES

To investigate the role of yeast elongation factor 3 (eEF3) in IRES translocation, 500 nM eEF3-ATP, Cy5-60S, and 10 or 200 nM eEF2-GTP were co-delivered to immobilized 40S–CrPV complexes. A kinetics analysis revealed that the rates of both forward and reverse rotations were unaffected by the presence of eEF3 (Supplementary Figure S8). FRET intensity distributions were similarly unaffected. It is possible that eEF3 functions rapidly at timescales faster than the time resolution of our experiments (100 ms). To examine this possibility, we repeated the experiments outlined above in the presence of the non-hydrolyzable ATP analogue, ADPNP. It stabilizes eEF3 on the ribosome and prevents the L1 stalk opening by eEF3 (49,89). Similarly, rotation rates and the FRET intensity remained unchanged (Supplementary Figure S8). Thus, we concluded that eEF3 has no effects on the translocation of the 80S–CrPV IRES complex.

## DISCUSSION

Recent structures of 80S–IRES-complexes mapped a structural pathway of translocation. However, the dynamic motions of IRES-driven translation have not been directly observed. Here, we followed the intersubunit conformation of eukaryotic ribosomes during initiation and translocation of CrPV IRES in real-time. We assigned the high (0.34) FRET state to the non-rotated ribosomes and the low (0.22) FRET state to the semi-rotated ribosomes, based on Cryo-EM structures of pre-translocation and post-translocation (17,54) 80S–CrPV IRES complexes. Pre-translocation 80S–IRES complexes are conformationally dynamic and spontaneously exchange between the semi-rotated and non-rotated states. This directly confirms a hypothesis, based on Cryo-EM structures, that 80S–CrPV IRES complexes sample different rotational conformations (17,18). Importantly, 80S–IRES complexes are kinetically heterogeneous, as spontaneous exchange was best described by double exponential fits. Furthermore, there were two populations of ribosomes. About 80% of the pre-translocation complexes were predominantly occupying the semi-rotated state, while 20% of the pre-translocation complexes mainly occupied the non-rotated state (Supplementary Figure S9). This implies that the system is not at a simple two state equilibrium and additional, unobserved intermediates might exist.

Spontaneous rotations in pre-translocation 80S–IRES complexes resemble the behavior of tRNA pre-translocation complexes, thus indicating similarities between the two types of translocation. The rate of spontaneous rotations in pre-translocation 80S–IRES complexes is comparable to that of bacterial pre-translocation ribosomes complexed with tRNA, as measured by smFRET studies (46,90,91). When eEF2 binds to the 80S–IRES complex, it transiently places the ribosome into the rotated conformation. One point of contention is whether eEF2 can bind to the non-rotated conformation or whether eEF2 binding captures the rotated state of the 80S–IRES complex. The ability of translocase to interact with non-rotated ribosomes is well described. EF-G sampling of non-rotated ribosomes with empty A-sites was detected by single-molecule fluorescence (43) and visualized by Cryo-EM (92). L11-tRNA (33,93) and S6-L9 FRET (91) indicate that EF-G engages both rotated and non-rotated ribosomes. The ensemble measurements of translocation kinetics argue that EF-G can engage both intersubunit conformations (94). Similarly, eEF2 also can recognize non-rotated ribosomes. eEF2-GDPNP causes a single nucleotide toeprint shift in the 5′ direction during post-translocation in non-rotated ribosomes (95). At a high concentration of E-site tRNA, eEF2 can promote reverse translocation, a reaction in which a factor is also expected to engage non-rotated ribosomes (96). Our kinetics analysis showed eEF2 promoted both reverse and forward subunit rotations in a concentration dependent manner, which also suggests eEF2 can efficiently recognize both semi-rotated and non-rotated ribosomes. Together, these results demonstrate that the ability of translocase to interact with non-rotated ribosomes is conserved between prokaryotic and eukaryotic elongation, as well as in tRNA and IRES translocation.

### eEF3 in IRES translocation

The lack of eEF3 effects on CrPV translocation are in line with our understanding of eEF3 function. During tRNA translocation, the L1 stalk contacts the elbow of the P-site tRNA. Post-translocation eEF3 opens the L1 stalk, thus allowing E-site tRNA dissociation. In the pre-translocation 80S–IRES complex, the domain I of CrPV IRES displaces the L1 stalk, placing it in a position that is more open than in the tRNA pre-translocation complex (16). The translocated IRES pushes the L1 stalk open even further, beyond to what is found in post-translocation ribosomes complexed with tRNA (54). This movement of the L1 stalk is a result of outward movements of domains I and II from the E-site that occur concurrently with reverse head swiveling during the late stages of IRES translocation (18). Thus, opening of the L1 stalk is a part of IRES translocation, which explains why eEF3 had no effect on the dynamics of intersubunit rotations and, congruently, had no effect on the recruitment of the first tRNA (65).

### Role of eEF2 in IRES translocation

In the presence of eEF2 and GTP, 80S–CrPV IRES complexes repeatedly translocated and back translocated. Ribosomes became static after eEF2 was washed off, indicating that repeated translocation events were due to multiple rounds of eEF2-ribosome interactions. Both reverse and forward rotations are promoted by eEF2 and require GTP hydrolysis (at least for forward translocation), as eEF2-GDP had only modest effect on ribosome conformation (Supplementary Figure S6). In prokaryotes, translocation in the presence of GDPNP is slower than in the presence of GTP (42,43). Similarly, <20% of 80S–IRES–eEF2 complexes underwent translocation in the presence of GDPNP, as most ribosomes were stabilized in the fully rotated state (Figures 3B and 5A). Thus, eukaryotic IRES translocation is remarkably similar to tRNA translocation.

The Brownian mechanism of translocation has been long proposed (97,98). There, the movement occurs stochastically due to Brownian forces and directionality is provided by rectifying energy. It is supported by a number of studies that showed translocation and conformational rearrangements associated with translocation can occur spontaneously (reviewed in (99,100)). Structures of translocation intermediates show that EF-G/eEF2 extends into the A-site of the ribosome. This suggests that translocase acts as a ‘pawl', preventing reverse sliding of the tRNA. Our results show that the ribosome spontaneously translocated and back translocated in the presence of eEF2 and sordarin. Neither reaction required GTP hydrolysis nor a phosphate release, as shown by wash experiments. The spontaneous and rapid nature of these reactions indicates that sordarin captures the ribosome in the unlocked state, whereas IRES translocation occurs over a flat energy landscape, as previously suggested (52). Remarkably, after the first somewhat slower translocation event in the presence of GDP and sordarin, subsequent rounds of translocation were as fast as translocation in the presence of GTP (Supplementary Figure S7). This suggests that GTP hydrolysis is used to achieve the unlocked state. Once the ribosome is unlocked, IRES translocation is thermally driven in the presence of the drug. Here, eEF2 acts as a classical enzyme that decreases the energy barrier of translocation making possible for it to occur rapidly due to low energy Brownian fluctuations. The release of rectifying energy gives translocation directionality. The ribosome being unlocked during intersubunit rotation suggests that release of rectifying energy occurs after intersubunit rotation and is possibly associated with the dissociation of eEF2 and/or final positioning of tRNA with polypeptide chain in the P-site. Together, these results support the Brownian mechanism by directly showing that the mid and late stages of translocation are thermally driven in the presence of sordarin.

CrPV IRES mimics tRNA and translation factors. Despite the ability of the IRES to trigger conformational changes in the ribosome associated with initiation and elongation, the dynamics and conformation of the 80S–IRES pre-translocation complex differ from that of tRNA translocation. Further investigation of the dynamics of eukaryotic tRNA translocation, specifically high-resolution structures of the 80S-tRNA complex with eEF2 and sordarin, will be needed to see if tRNA translocation is thermally driven by the eEF2-sordarin complex.

## Supplementary Material

gkad476_Supplemental_Files

## Data Availability

The data underlying this article will be shared on reasonable request to the corresponding author.
