# Supplementary material for: Sordarin bound eEF2 unlocks spontaneous forward and reverse translocation on CrPV IRES
Source: Nucleic Acids Res. 2023 Jun 7;51(13):6999–7013. doi: 10.1093/nar/gkad476 (PMC10359634; doi:10.1093/nar/gkad476)
Supplement: gkad476_Supplemental_Files [file gkad476_supplemental_files.zip › Supplementary Tables Legends.docx]

**Supplementary table legends**

Supplementary Table 1. Primers used in this study.

Supplementary Table 2. Dynamics of 80S IRES complexes in the absence of eEF2.

A. 60S subunit arrival rates. Related to Fig. 1C and Supp. Fig. 2A.

B. Frequency of spontaneous intersubunit rotations. Related to Fig. 2C and Supp. Fig. 4.

C. Kinetics of reverse spontaneous intersubunit rotations. Related to Fig. 2C and Supp. Fig. 4.

D. Kinetics of forward spontaneous intersubunit rotations. Related to Fig. 2C and Supp. Fig. 4.

Supplementary Table 3. Kinetics of intersubunit rotation in the presence of eEF2.

A. Kinetics of reverse intersubunit rotations. Related to Fig. 3C, 4C and Supp. Fig. 5.

B. Kinetics of forward intersubunit rotations. Related to Fig. 3C, 4C and Supp. Fig. 5.

Supplementary Table 4. Kinetics of intersubunit rotation in the presence of eEF2 and sordarin.

A. Kinetics of reverse intersubunit rotations. Related to Fig. 4.

B. Kinetics of forward intersubunit rotations. Related to Fig. 4.

Supplementary Table 5. Frequency and kinetic parameters of intersubunit rotations.

A. Frequency of intersubunit rotations with GTP, GDP and GDPNP in the presence or absence of sordarin. Related to Fig. 5A.

B. Kinetic parameters of the first intersubunit rotation in the presence of sordarin, GTP and GDP. Related to Fig. 5B

C. Rotations per trace in 30 seconds time window in "wash-off" experiments. Related to Fig. 6

Supplementary Table 6. Strains used and generated in this study.
