# Supplementary material for: Sordarin bound eEF2 unlocks spontaneous forward and reverse translocation on CrPV IRES
Source: Nucleic Acids Res. 2023 Jun 7;51(13):6999–7013. doi: 10.1093/nar/gkad476 (PMC10359634; doi:10.1093/nar/gkad476)
Supplement: gkad476_Supplemental_Files [file gkad476_supplemental_files.zip › Supplementary_material.pdf]

SUPPLEMENTARY MATERIAL

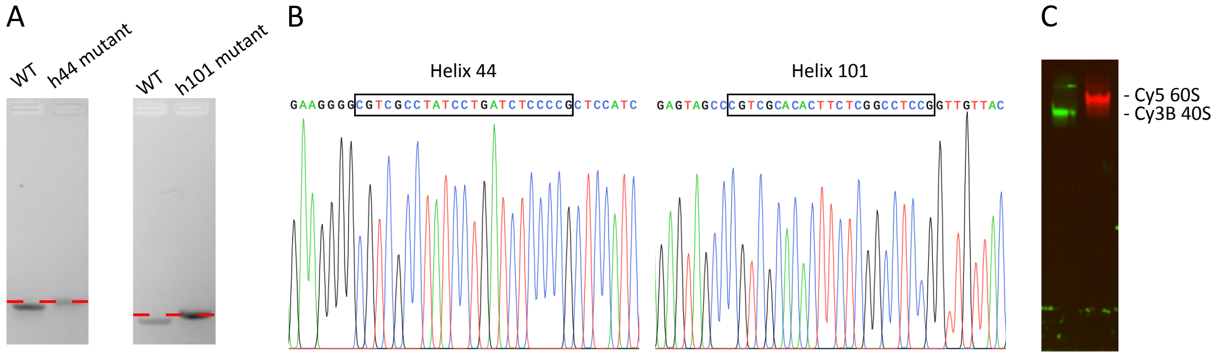

Supplementary Figure 1. Labeling of the yeast ribosomal subunits. Hairpins were introduced into phylogenetically variable regions of rRNA. The sp68 hairpin was inserted into the 18S rRNA, replacing 1699-1702 nucleotides, thus extending the helix 44. The sp22 hairpin was inserted into the 25S rRNA, where it replaced nucleotides 3351-3352, thus extending the helix 101. To generate yeast strains carrying the mutated RDNA operon as the sole source of rRNA, a plasmid bearing the mutated RDN operon was transformed into the yeast and the loss of the wildtype RDN operon carrying plasmid was performed, as described in the Materials and Methods section. A. The presence of the mutated RDN operon was confirmed by PCR, with primers flanking either the helix 44 or helix 101 regions. The PCR product was ~19 and 21 nts longer for the mutant hairpins. B. The PCR product was sequenced to further confirm the absence of contamination by the wildtype ribosomal DNA. The box indicates the inserted hairpin. C. The labeling and integrity of ribosomal subunits were confirmed by composite agarose-polyacrylamide electrophoresis, where purified and labeled ribosomal subunits migrated as a single band. The labeling efficiency was determined spectrophotometrically and was found to be ~92% for the 40S subunits and ~88% for the 60S subunits.

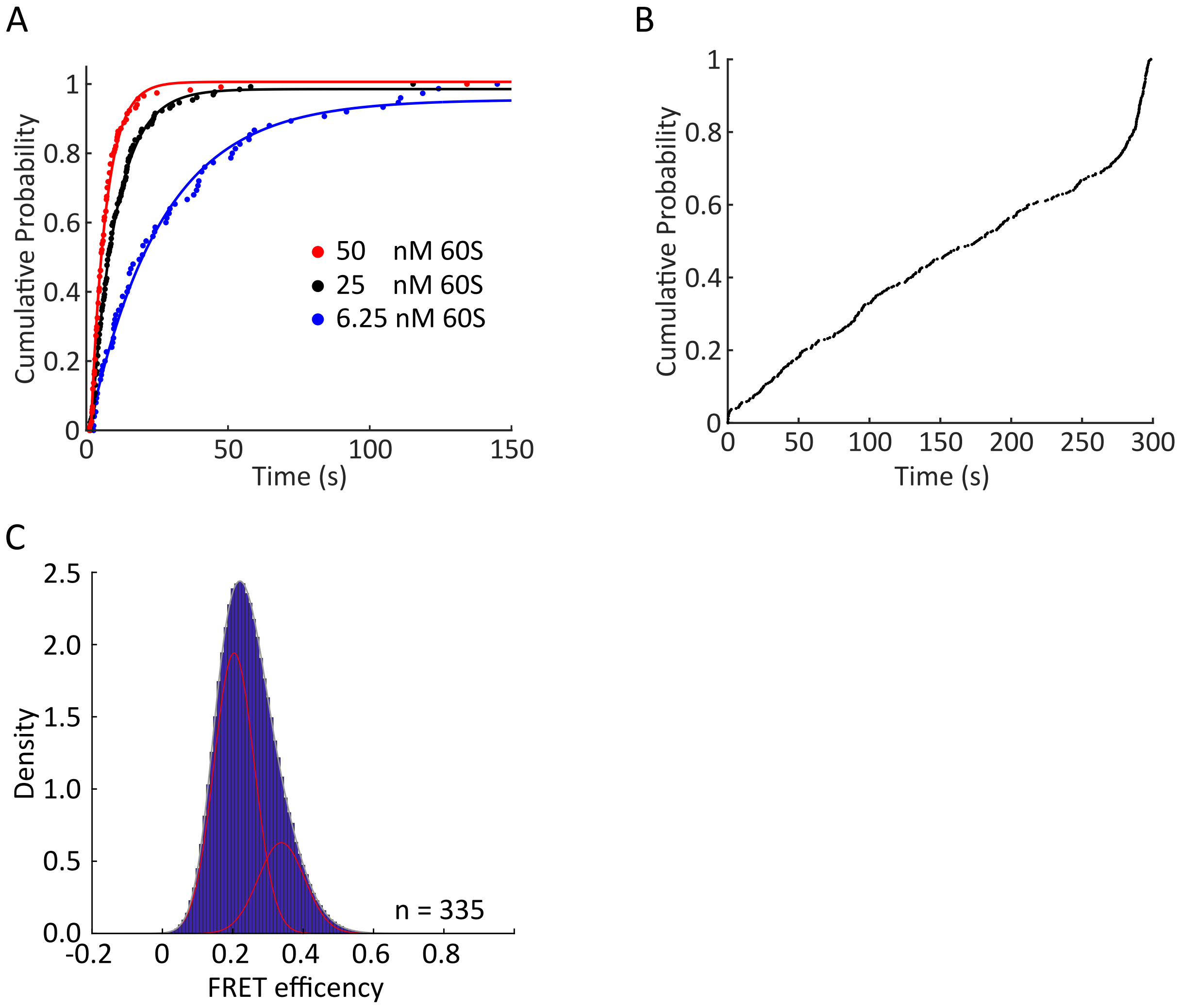

Supplementary Figure 2. 60S subunit arrival to the 40S-CrPV IRES complex. A. The cumulative probability of arrival times for 6.25, 25, and 50 nM 60S ribosomal subunits. The empirical Cumulative Distribution Functions were fitted with the single exponential model, ($y = A_0(1 - \exp(-k_{obs}/t)) + \mathrm{c}$, n = 102, 130, and 117 for 6.25, 25, and 50 nM, respectively). B. FRET lifetimes of 60S ribosomal subunit delivery experiment. Hockey stick-like appearance is due to the FRET signal lasting until the end of the movie (n = 335 molecules). C. The FRET distribution was single modal with a minor right shoulder, indicating that ribosomes prefer a low FRET state. The double Gaussian fit estimated FRET efficiency means were 0.2 ± 0.06 (95% CI) for the low FRET state and 0.33 ± 0.07 (95% CI) for the high FRET state (n = 335 molecules).

A

PDBID	Complex description	Body rotation	Distance	Head rotation	PKI position
5JUU	eEF2-GDP-Sordarin-TSV IRES bound 80S, least rotated	0.5°	78.3 Å	1°	P
5JUT	eEF2-GDP-Sordarin-TSV IRES bound 80S, almost NR	1°	79.3 Å	14°	A/P
3J6Y	TSV IRES bound 80S, Class I	2°	79.8 Å	8°	A
5JUP	eEF2-GDP-Sordarin-TSV IRES bound 80S, mid-rotated	5°	81 Å	17°	A/P
5JUS	eEF2-GDP-Sordarin-TSV IRES bound 80S, mid-rotated	5°	81.5 Å	17°	A/P
3J6X	TSV IRES bound 80S, Class II	5°	83 Å	8°	A
5JUO	eEF2-GDP-Sordarin-TSV IRES bound 80S, fully rotated	10°	91.1 Å	12°	A/P

B

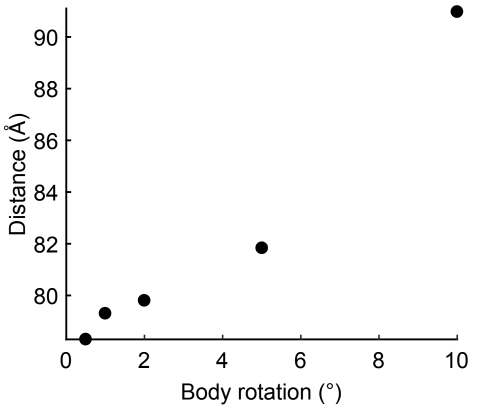

Supplementary Figure 3. Interpretation of the FRET states. A. Estimated distances between the two labeling sites on helix 44 of 18S rRNA and helix 101 of 25S rRNA were measured as a distance from C4 of the ribose of G1698 to C4 of the ribose of C3350, based on published yeast 80S structures. The structures of helix 44 and helix 101 are not fully resolved in pre-translocation 80S-CrPV IRES complexes. Thus, we used the structures of the related pre-translocation 80S-TSV IRES complexes to measure the distances. B. The relationship between intersubunit conformation and the distance between h44 and h101.

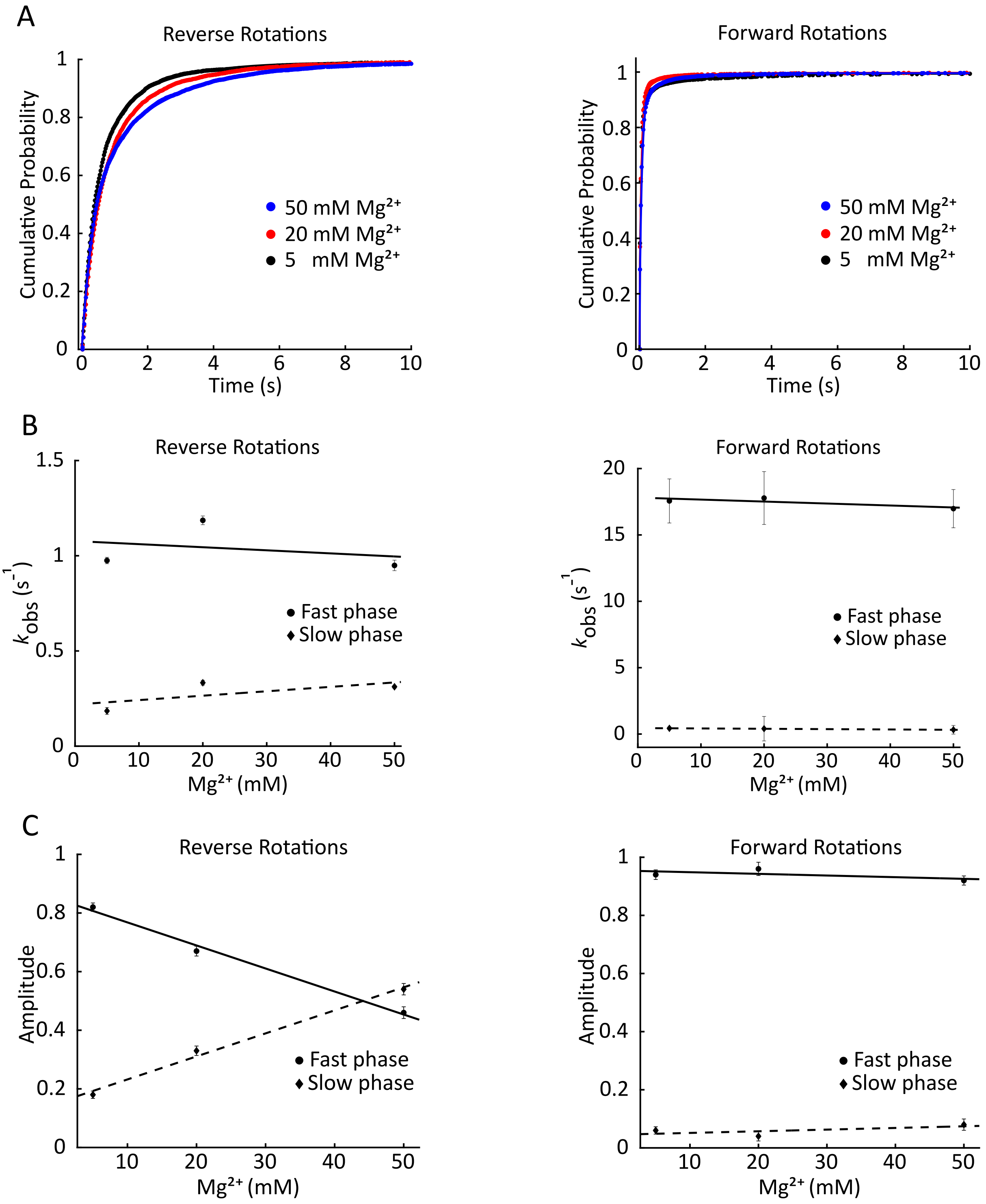

Supplementary Figure 4. Spontaneous rotations in pre-translocation 80S-CrPV IRES complexes resolved at 25 ms exposure time. A. Cumulative probability plot showed the comparison of reverse (left) and forward rotation (right) dwell times at different magnesium concentrations. Both are best approximated by double exponential fits. B. The rates of reverse (left) and forward rotations (right) show insignificant changes with increased Mg^{2+} concentration. C. Ribosomes prefer the rotated state at elevated Mg^{2+} due to the slow-down of reverse rotation, which is manifested by a relative change of amplitudes for the fast and slow phases. The amplitudes of the double exponential fit for reverse (left)

and forward rotations (right) at different Mg^{2+} concentrations. The amplitude of the fast phase for reverse rotations decreases with the increase of Mg^{2+} concentration (n = 198, 224, and 214 for 5 mM, 20 mM, and 50 mM Mg^{2+}, respectively).

A

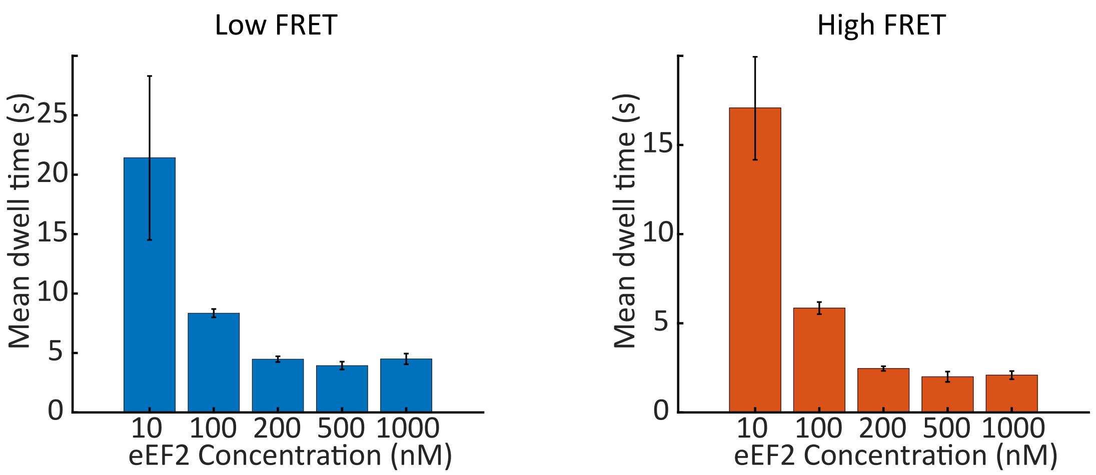

Supplementary Figure 5. Kinetics of eEF2-induced intersubunit rotations. A. Mean dwell times of the low FRET state (rotated ribosomes, left panel) and the high FRET state (non-rotated ribosomes, right panel) at different eEF2 concentrations. Dwell times decreased at higher eEF2 concentrations.

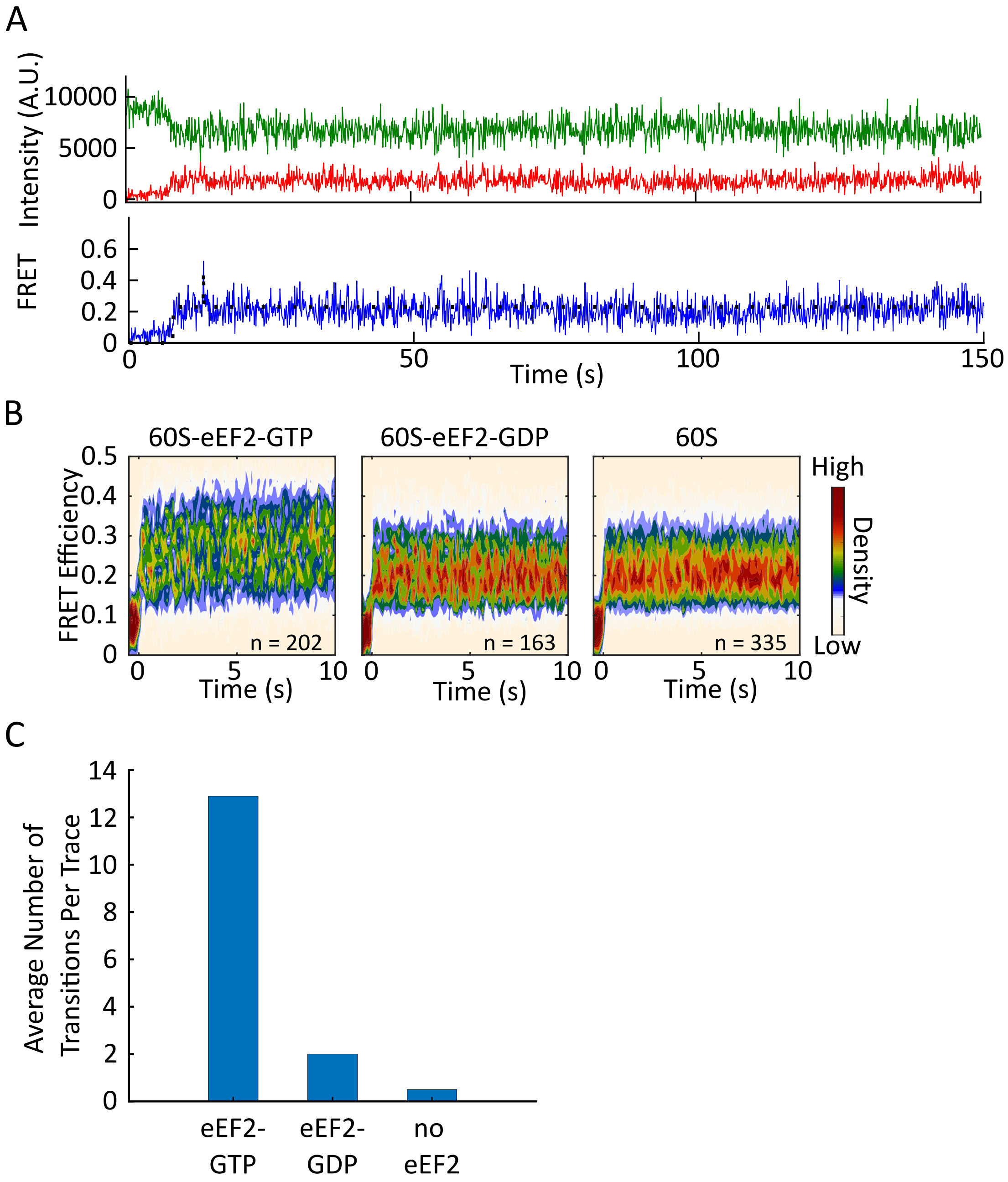

Supplementary Figure 6. eEF2-GDP has modest effects on ribosome conformations. A. Example trace in the presence of eEF2-GDP. B. eEF2-GDP has no influence on global FRET efficiency. Post-synchronization plot of FRET efficiency, synchronized to 60S ribosomal subunit arrival (zero time point). C. Average number of transitions per trace.

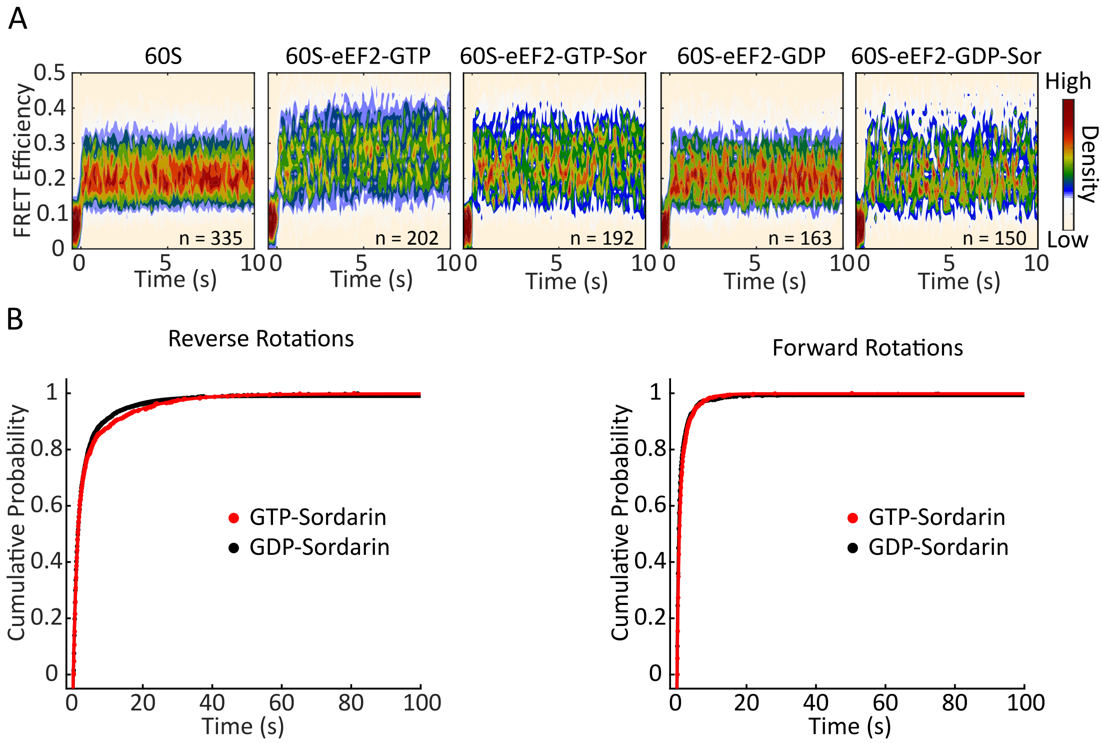

Supplementary Figure 7. GTP is not required for subunit rotations in the presence of sordarin. A. Post-synchronization plot of FRET efficiency, synchronized to 60S ribosomal subunit arrival (zero time point). B. After the first conformational change, there are no differences between GTP and GDP in the presence of sordarin. Cumulative probability plot of dwell times of all subsequent reverse (left) and forward rotations (right).

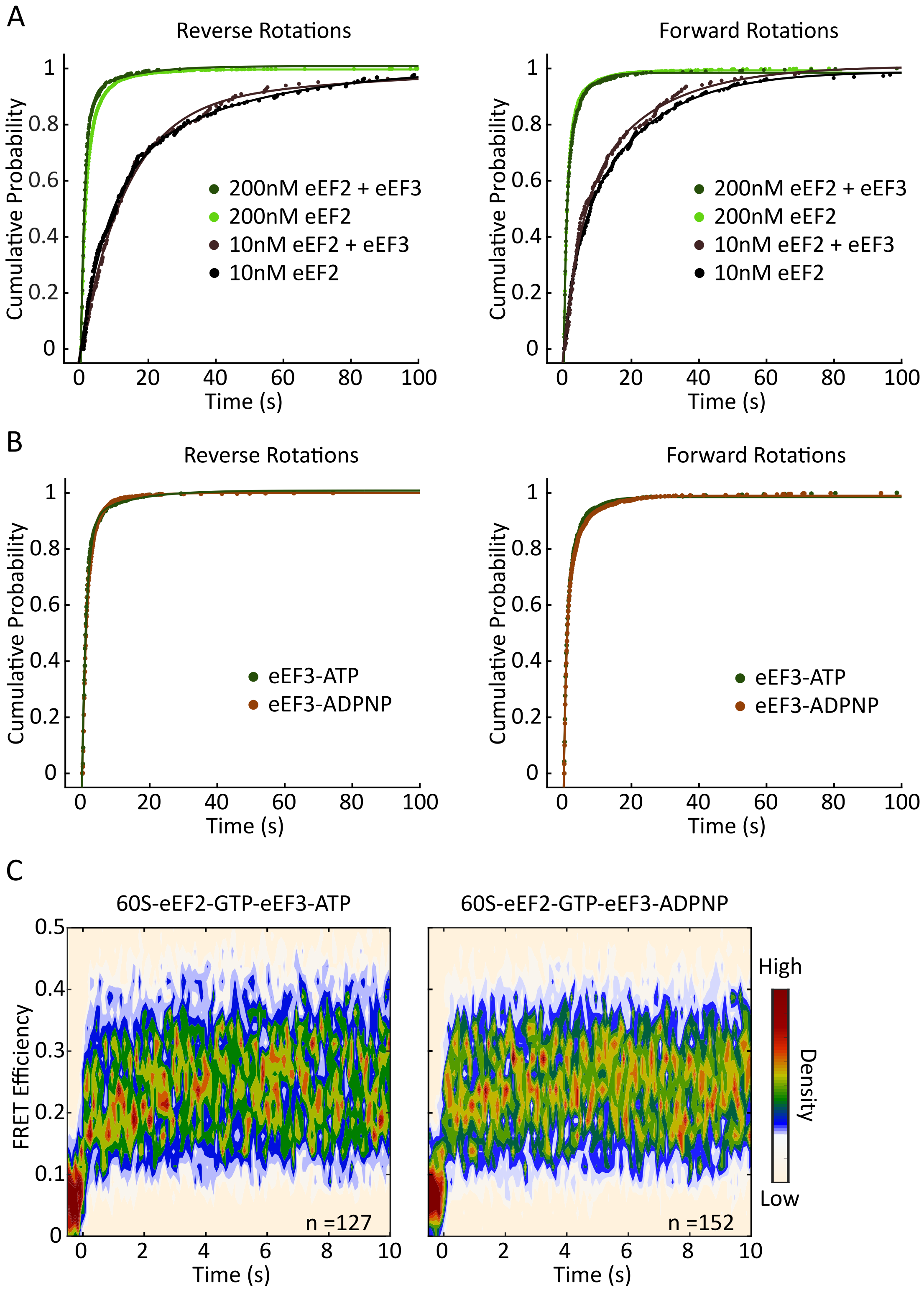

Supplementary Figure 8. eEF3 does not affect intersubunit dynamics. A. Cumulative probability plot shows a comparison of reverse (left) and forward rotation (right) dwell times with and without eEF3. B. Cumulative probability plot shows a comparison of reverse (left) and forward rotation (right) dwell times with the presence of ATP and ADPNP (n = 127 and 152, respectively). C. Post-synchronization plot of FRET efficiency observed before and after 60S subunit arrival (n = 127 and 152, respectively).

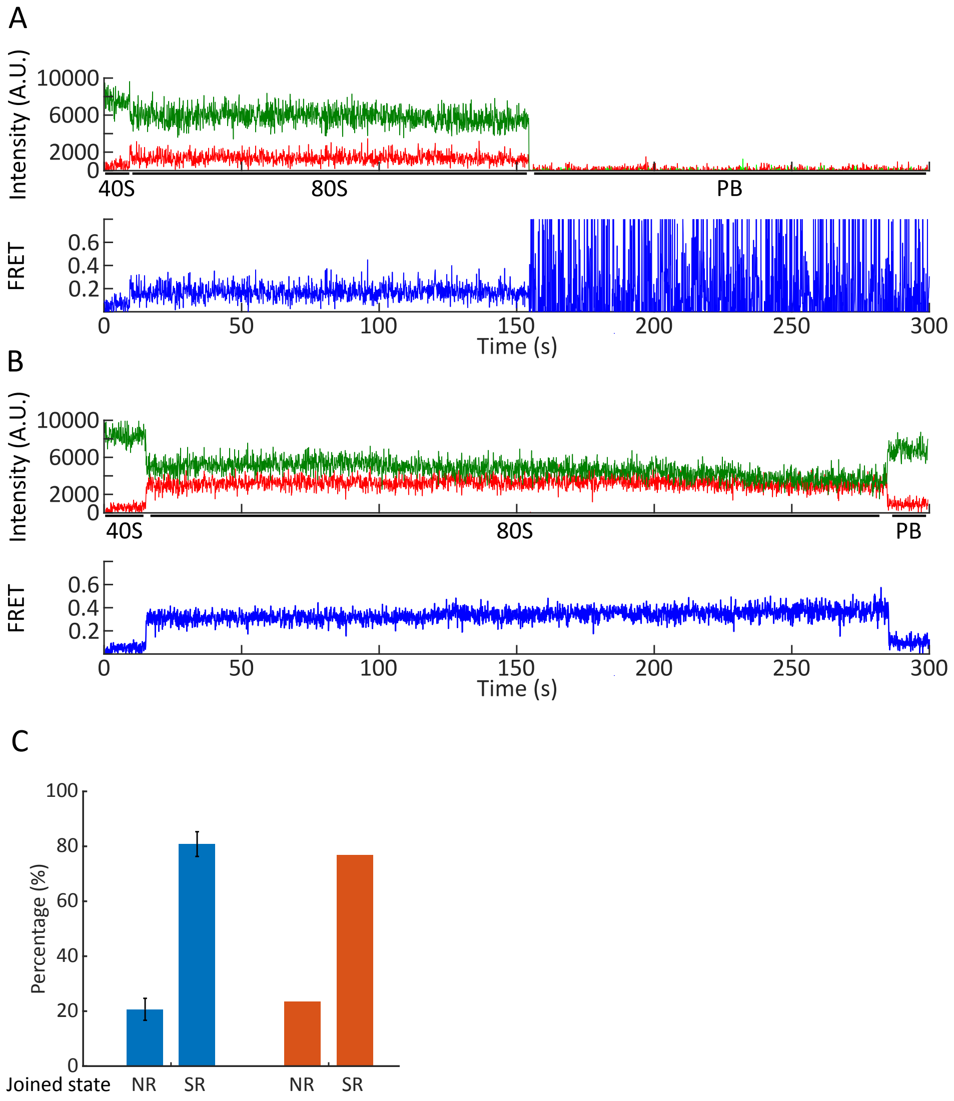

Supplementary Figure 9. Conformational heterogeneity of pre-translocation 80S-CrPV IRES complexes. A. Example trace showing the ribosome that joined and remained in the semi-rotated state. Spontaneous rotations are masked by camera averaging due to the 100 ms exposure time. B. Example trace showing the ribosome that joined and remained in the high FRET state. C. Percentage of traces that joined in the non-rotated or rotated state (blue, error bars are 95% CI) and relative fractions from the double Gaussian fit of FRET efficiency (red).
